# Supplementary material for: Community Complexity Does Not Weaken Pairwise Coevolution in a Soil Bacterial Community
Source: Ecol Lett. 2025 Nov 11;28(11):e70260. doi: 10.1111/ele.70260 (PMC12605764; doi:10.1111/ele.70260)
Supplement: Supplementary file 1 — Appendix S1: ele70260‐sup‐0001‐Supinfo.docx. [file ELE-28-0-s001.docx]

**Estimating transfer size**

Transfer size (density) was estimated from density data from the mono- and coculture treatments at the end of week 10. Colony counts used for the estimates are presented in table 1 below. Density was calculated using the 35 µl volume plated following the equation: Cell density = nb CFU * 10^dilution used^ / volume plated.

Table 1 Colony counts for Pseudomonas and Variovorax in the mono- and coculture treatment at the end of week 10.

| treatment | rep | dilution | P | V | density CFU/ml | density CFU/ml |
| --- | --- | --- | --- | --- | --- | --- |
| P_mono | 2 | 5 | 37 | NA | 1.1E+08 | NA |
| P_mono | 5 | 5 | 37 | NA | 1.1E+08 | NA |
| P_mono | 6 | 5 | 24 | NA | 6.9E+07 | NA |
| P_mono | 7 | 5 | 25 | NA | 7.1E+07 | NA |
| P_mono | 8 | 5 | 32 | NA | 9.1E+07 | NA |
| V_mono | 3 | 5 | NA | 39 | NA | 1.1E+08 |
| V_mono | 5 | 5 | NA | 39 | NA | 1.1E+08 |
| V_mono | 6 | 5 | NA | 39 | NA | 1.1E+08 |
| V_mono | 7 | 5 | NA | 54 | NA | 1.5E+08 |
| V_mono | 8 | 5 | NA | 42 | NA | 1.2E+08 |
| PV_co | 1 | 5 | 33 | 21 | 9.4E+07 | 6.0E+07 |
| PV_co | 2 | 5 | 27 | 18 | 7.7E+07 | 5.1E+07 |
| PV_co | 3 | 5 | 19 | 26 | 5.4E+07 | 7.4E+07 |
| PV_co | 4 | 5 | 25 | 24 | 7.1E+07 | 6.9E+07 |
| PV_co | 5 | 5 | 22 | 36 | 6.3E+07 | 1.0E+08 |
| PV_co | 6 | 5 | 26 | 20 | 7.4E+07 | 5.7E+07 |
| PV_co | 7 | 5 | 15 | 20 | 4.3E+07 | 5.7E+07 |
| PV_co | 8 | 5 | 20 | 21 | 5.7E+07 | 6.0E+07 |

**Justification for removing count data for timeshift assay of coculture evolved *Pseudomonas* (6 weeks) and *Variovorax* (10 weeks) (data point VD4PC4)**

We observed an atypically high colony count of *Variovorax* (111) combined with a low count of *Pseudomonas* (4) in this one replicate. The same 10-week coculture evolved *Variovorax* produced 38 and 39 colonies when cultured with the other timepoints of *Pseudomonas.* Furthermore, this *Variovorax* is unusually high even when compared to the whole dataset (Fig S1A). We suspect that this is due to a wet lab error (either when setting up the timeshift experiment, or when plating the result i.e. lack of vortexing resulting in biofilms not being resuspended). We decided to remove this datapoint before carrying out any analysis, therefore it was not the case that this datapoint did not fit our results. Importantly, this one replicate out of the 8 from the same condition (6-week P, 10-week V, Coevolution, Fig S1B) has high influence on the outcome of the analysis as presented below:


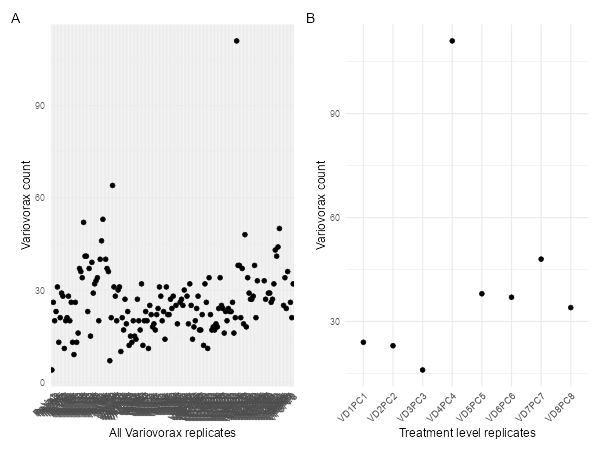


*Figure S1 Variovorax counts of all replicates in the dataset (A) and replicates within the treatment levels (6-week P, 10-week V, Cocolture) highlighting the atypical count for Variovorax in VD4PC4 (colony count for the combination of Pseudomonas (6-week) and Variovrax (10-week) in the timeshift experiment.*

Analysing coevolution within treatments, including VD4PC4 counts, makes the case for coevolution between *Pseudomonas* and *Variovorax* much stronger (GLMM: *Pseudomonas* time x *Variovorax* time: χ2_3_ = 14.29, p < 0.01) as opposed to when excluding it (GLMM: *Pseudomonas* time x *Variovorax* time: χ2_3_ = 7.15, p = 0.067, as reported in manuscript) driven by a much larger effect size (-0.45 vs -0.11, effect sizes represent change in proportion P on log scale) at the corresponding timepoint combinations (6-week P, 10-week V).

When looking at how community complexity affects coevolution, including VD4PC4 counts leads to a significant 3-way interaction between P_time x V_time x treatment (GLMM: *Pseudomonas* time x *Variovorax* time x complexity interaction χ^2^_3_ = 7.87, p = 0.049) that completely disappear if VD4PC4 is removed from the analysis (GLMM: *Pseudomonas* time x *Variovorax* time x complexity interaction χ^2^_3_ = 3.54, p = 0.32). This once again is driven by a larger effect size at the 6-week P 10-week V combination (0.82 vs 0.41, effect sizes represent change in proportion P on log scale). Based on this, we thought that including this observation, that could likely have resulted from a wet-lab error, would be inflating effect sizes of coevolution and would draw conclusions that are not supported by the rest of the dataset. Consequently, we choose to keep this point excluded.

The code and dataset are made available with the submission, and we have updated this with a complete analysis where observation VD4PC4 is included.

We’ve added clarification in the manuscript at **line 178:** “(likely due to a wet-lab error) and was excluded from the dataset prior to analysis (for further details see supplementary data)” Added a reference to supplementary data containing the above explanation. Furthermore, a separate analysis in the form of an R script is provided where the whole analysis is carried out on the complete dataset including the outlier.
